# Factors affecting chiropractor requests for full‐length spinal radiography: A scoping review

**DOI:** 10.1002/jmrs.566

**Published:** 2022-01-07

**Authors:** Thomas R. Readford, Melanie Hayes, Warren Michael Reed

**Affiliations:** ^1^ Discipline of Medical Imaging Science, Sydney School of Health Sciences, Faculty of Medicine and Health The University of Sydney Camperdown New South Wales Australia; ^2^ Discipline of Work Integrated Learning, Sydney School of Health Sciences, Faculty of Medicine and Health The University of Sydney Camperdown New South Wales Australia; ^3^ Medical Imaging Optimisation and Perception Group (MIOPeG), Sydney School of Health Sciences, Faculty of Medicine and Health The University of Sydney Camperdown New South Wales Australia

**Keywords:** chiropractic, diagnostic radiography, full‐length spinal X‐ray

## Abstract

Chiropractors often refer their patients for full‐length (three‐ to four‐region) radiographs of the spine as part of their clinical assessment, which are frequently completed by radiographers in medical imaging practices. Overuse of spinal radiography by chiropractors has previously been reported and remains a contentious issue. The purpose of this scoping review was to explore the issues surrounding the utilisation of full‐length spinal radiography by chiropractors and examine the alignment of this practice with current evidence. A search of four databases (AMED, EMBASE, MedLine and Scopus) and a hand search of Google was conducted using keywords. Articles were screened against an inclusion/exclusion criterion for relevance. Themes and findings were extracted from eligible articles, and evidence was synthesised using a narrative approach. In total, 25 articles were identified, five major themes were extracted, and subsequent conclusions drawn by authors were charted to identify confluent findings. This review identified a paucity of literature addressing this issue and an underrepresentation of relevant perspectives from radiographers. Several issues surrounding the use of full‐length spinal radiography by chiropractors were identified and examined, including barriers to the adherence of published guidelines for spinal imaging, an absence of a reporting mechanism for the utilisation of spinal radiography in chiropractic and the existence of a spectrum of beliefs amongst chiropractors about the clinical utility and limitations of full‐length spinal radiography. Further investigation is required to further understand the scope of this issue and its impacts for radiation protection and patient safety.

## Graphical abstract

Australian chiropractors are able to refer their patients for full‐length spinal X‐rays. Overuse of spinal radiography by chiropractors has previously been reported and remains a contentious issue. This review explored the issues surrounding the use of full‐length spinal X‐rays by chiropractors and the alignment of this practice with current evidence

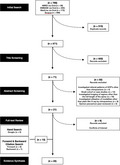

## Introduction

Chiropractors are registered health practitioners who perform manual therapies to treat a myriad of health conditions.^1^ Amongst the founding principles of chiropractic medicine was the belief that peripheral ailments of various aetiology are the result of a blockages of the body’s innate energy.^2^ These blockages are said to be caused by minute misalignments of the spinal column termed ‘chiropractic vertebral subluxations’ (CVS), which could reportedly be treated through manual adjustment and identified using spinal radiography.^3^, ^4^ It has been argued that beliefs such as these, which are historical in nature and do not reflect the contemporary understandings of physiology taught to chiropractors,^5^ have to some extent pushed chiropractic to the periphery of the broader Australian healthcare system.^6^ However, chiropractic remains a prominent health profession in Australia, with a substantial client base in the community.^1^

As registered health practitioners, Australian Chiropractors can refer their patients for diagnostic investigations, including radiological studies, based on appropriate clinical indications.^7^ Frequently, chiropractors refer their patients for full‐length spinal (FLS) X‐Rays, to assess for underlying pathological and structural changes which may have some impact upon their treatment.^4^, ^8^ Between 2014 and 2015, approximately 130,000 three‐ to four‐region spinal X‐rays were performed in Australia.^8^, ^9^ Most were requested by chiropractors.^8^, ^9^ In Australia, radiographers are often requested to complete FLS X‐ray examinations by chiropractors.^1^ In keeping with their professional responsibilities as health care practitioners, radiographers and referring chiropractors must exercise judgment in determining whether a requested examination is justified in the context of currently available evidence and the known risks of unnecessary diagnostic investigations.^7^, ^10^ There is conflicting evidence to support the continued use of FLS radiography in chiropractic,^5^, ^11^, ^12^ which in turn has made this issue a focal point for controversy in chiropractic academia.^4^

Therefore, the completion of chiropractor‐requested FLS X‐rays may present a challenge to the foundations of evidence‐based practice and should be considered an important issue with impacts for both radiographers and chiropractors.^5^, ^12^ The purpose of this scoping review was to explore the issues surrounding the utilisation of FLS radiography by chiropractors and examine the alignment of this practice with current evidence.

## Methods

### Protocol

The Preferred Reporting Items for Systematic Reviews and Meta‐Analyses – Scoping review extension (PRISMA‐ScR) checklist was used for this review.^13^ The methodology used was adapted from the proposed methodology for conducting a scoping review set out by Sucharew and Macaluso (2019).^14^

### Search strategy

An initial search was conducted in April 2021 using variations of the keywords ‘chiropractic’, ‘spinal manipulation’, ‘X‐ray’ and ‘radiograph’ in four databases: AMED, EMBASE, Medline and Scopus. A hand search of Google was conducted using the same parameters to identify grey literature. Titles and abstracts were then reviewed for eligibility by the authors, followed by a full‐text review of eligible articles. Forward and backward citation searching was implemented to identify additional eligible articles. Selected authors were also contacted individually for background information which was later used during evidence synthesis.

### Eligibility criteria

Articles were included if they investigated FLS X‐ray utilisation by chiropractors. Position statements from regulatory and professional bodies were also included in evidence synthesis. Search results were limited to articles published between 2010 and 2021 to ensure evidence reflected current attitudes and knowledge. Articles needed to address full‐length spinal radiography, defined as imaging which includes the cervical, thoracic, lumbar and/or sacral spine^8^, ^9^ to be eligible for inclusion in the data pool. Technical articles which investigated image quality and radiation dose in FLS X‐ray examinations were also included irrespective of referring discipline, as this was not deemed to be relevant in the context of establishing the limitations of the examination itself. Articles which specified that they examined the utilisation of FLS X‐Rays by practitioners of disciplines other than chiropractic were excluded, as were case reports, small cohort studies and opinion pieces. Articles where conflicts of interest or funding sources which may impact the validity of findings reported were excluded. Articles published in languages other than English were also excluded.

### Data charting process

Data were charted according to the alignment of eligible articles with key themes identified during a full‐text review. Data were charted by Investigator 1 (TRR), then reviewed by Investigators 2 and 3 (MH, WMR). Disagreements in the classification of data were resolved through discussion between investigators.

### Evidence synthesis

Conclusions drawn by authors were explored and described in the context of the themes extrapolated during the full‐text review of eligible articles. Issues surrounding the utilisation of FLS X‐Rays by chiropractors were explored and gaps in knowledge were described through a narrative approach.

## Results

**Figure 1:**
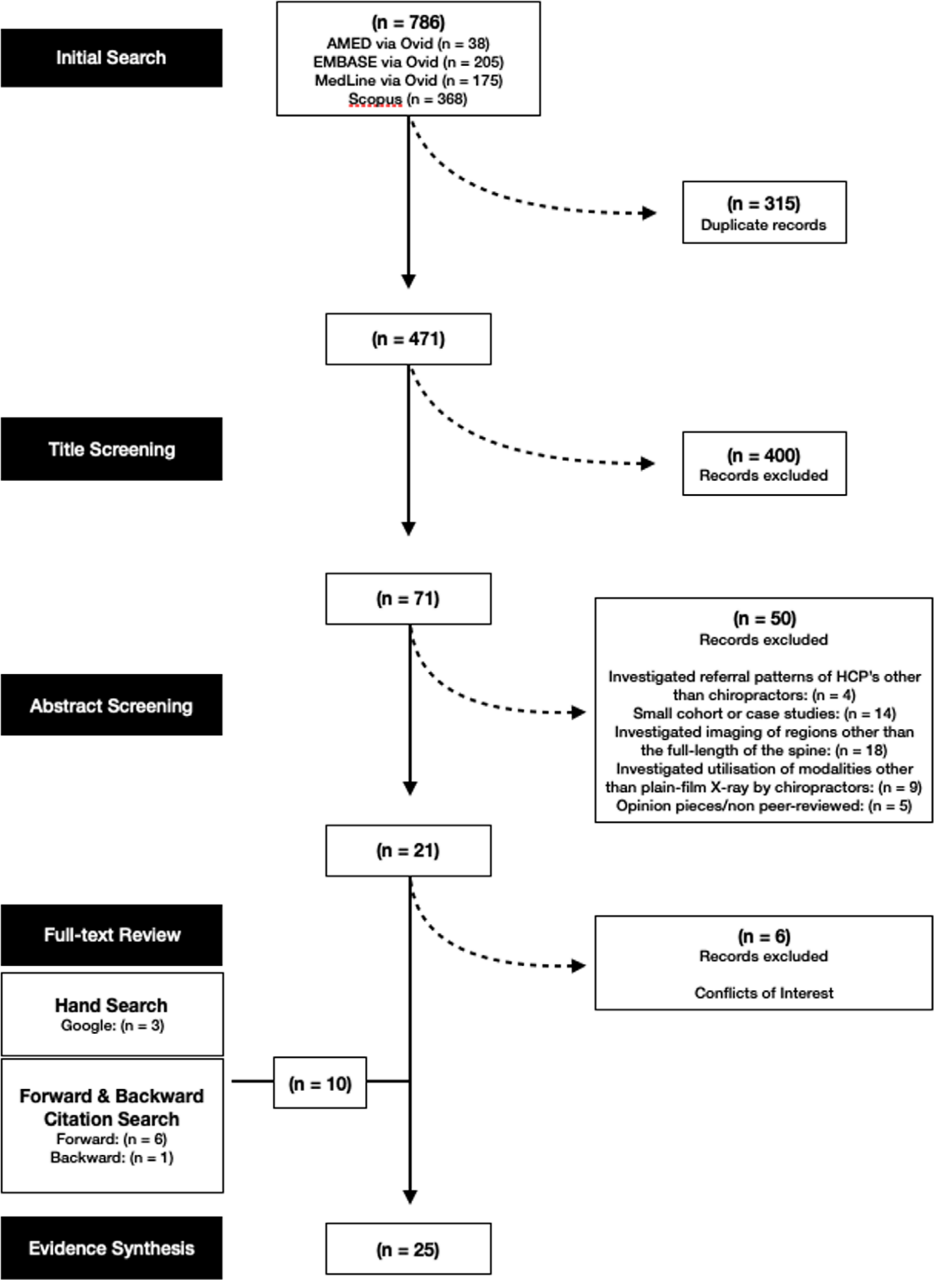
Flow diagram showing sources of data.

A total of 786 records were identified in the initial database search. After removing duplicates, 471 titles and abstracts were screened, with 71 full‐text papers assessed for eligibility. A total of 50 records were excluded for not meeting the inclusion criteria. Six records were excluded due to concerns raised by the authors about declared funding sources, which were perceived to be a source of potential bias. Ten additional articles were found through hand searching and forward and backward citation searching. In total, 25 eligible articles were included in the review (*n* = 25). A flow diagram showing sources of data is included as Figure 1.

### Study characteristics

A total of 21 of the 25 eligible articles (84%) examined in this review were written by authors with a background in chiropractic.^2^, ^3^, ^4^, ^5^, ^6^, ^11^, ^12^, ^15^, ^16^, ^17^, ^18^, ^19^, ^20^, ^21^, ^22^, ^23^, ^24^, ^25^, ^26^, ^27^, ^28^ A total of 11 of the eligible articles were reviews of existing literature, which examined evidence surrounding the use of FLS X‐rays in chiropractic in isolation, or as a part of wider studies examining the utilisation of medical imaging by chiropractors.^2^, ^3^, ^4^, ^5^, ^6^, ^12^, ^19^, ^24^, ^25^, ^28^ Seven cross‐sectional studies were identified, which investigated knowledge of and adherence to established guidelines for the utilisation of medical imaging, including FLS X‐rays in chiropractic and explored how knowledge of these guidelines impacted imaging referral patterns.^11^, ^16^, ^17^, ^18^, ^21^, ^26^, ^27^ Populations and perspectives were regionally diverse, with predominant author representation from Australia, North America and Europe. Our review was not able to identify any literature authored by radiographers which addressed chiropractor referrals for FLS radiography, which could be indicative of the wider underrepresentation of relevant perspectives from radiographers in discussions surrounding this issue.

### Thematic extraction

There were five major themes present throughout the reviewed articles; *(1) The historical integration of FLS radiography in chiropractic, (2) Clinical indications for FLS radiography in chiropractic, (3) Risks associated with FLS radiography, (4) Chiropractic techniques which prescribe the use of FLS radiography* and *(5) Current trends in the utilisation of FLS radiography in chiropractic*. The alignment of the findings of individual articles with different themes is explored in Table 1.

**Table 1:** Characteristics of included studies.

ID Author, Year	Region	Classification/ Study Design	Discipline of Lead Author	Relevant theme(s)				
				1. The historical Integration of FLS radiography in chiropractic	2. Indications for FLS radiography	3. Risks associated with FLS radiography (see Table 2)	4. Chiropractic techniques which prescribe the use of FLS radiography	5. Current trends in the utilisation of FLS radiography by chiropractors
Alcantara, 2010^15^	Canada	Cross‐sectional descriptive survey	Chiropractor				•	
Bussieres, 2010^16^	Switzerland	Randomised trial interventional study with postal follow‐ups	Chiropractor		•			
Bussieres, 2013^18^	USA	Cross‐sectional retrospective analysis	Chiropractor		•			•
Bussieres, 2014^17^	USA	Interrupted time series analysis	Chiropractor		•			•
Coleman, 2011^20^	USA	Simulated technical study	Chiropractor			•		
Coleman, 2013^19^	USA	Historical review	Chiropractor	•			•	
Corso, 2020^12^	Canada	Rapid review	Chiropractor		•	•	•	•
De Carvalho, 2021^21^	Canada	Cross‐sectional survey	Chiropractor					•
Department of Health, 2017^8^	Australia	Government publication	N/A					•
Harrison, 2018^4^	USA	Review	Chiropractor		•	•	•	•
Jenkins, 2016^11^	Australia	Cross‐sectional survey	Chiropractor		•	•	•	•
Jenkins, 2018^5^	Australia	Narrative Review	Chiropractor		•	•	•	•
Johnson, 2019^22^	USA	Editorial	Chiropractor	•				•
Law, 2016^29^	Hong Kong	Simulated technical study	Radiologist			•		
MBS Review Taskforce, 2016^9^	Australia	Professional Consultation Committee report	Multidisciplinary committee			•		•
Mogaadi, 2012^30^	Tunisia	Retrospective quantitative analysis	Biologist			•		
Oakley, 2020^3^	USA	Review	Chiropractor		•	•	•	•
Simpson, 2019^6^	Australia	Historical review	Chiropractor	•				•
Walker, 2011^23^	Australia	Cross‐sectional survey	Chiropractor		•			•
Young, 2014 (I)^24^	Australia	Historical review	Chiropractor	•			•	
Young, 2014 (II)^25^	Australia	Historical review	Chiropractor	•			•	
Young, 2016^2^	Australasia, North America, UK, Europe	Historical review	Chiropractor	•			•	•
Young, 2017 (I)^26^	Australasia, North America, UK, Europe	Survey	Chiropractor	•			•	
Young, 2017 (II)^27^	Australia	Thematic analysis	Chiropractor	•			•	
Young, 2019^28^	Australia	Historical review	Chiropractor	•			•	

#### Theme one – The historical integration of FLS radiography in chiropractic

Nine eligible articles addressed the historical integration of FLS radiography in chiropractic.^2^, ^6^, ^19^, ^22^, ^24^, ^25^, ^26^, ^27^, ^28^ Articles outlined the early uses of FLS radiography or ‘spinography’ by chiropractors to attempt to confirm vertebral subluxation theory. CVS was first described by chiropractic’s founder, D.D. Palmer, in 1895 as a minute misalignment of the vertebrae of the spine, which in turn could impinge a nerve and interrupt the flow of vital force, a concept derived from a central belief in innate intelligence and vitalism. Palmer termed these misalignments to be ‘subluxations’, appropriating established medical terminology for the displacement of a joint from its normal physiological position. Today, CVS as defined by Palmer has yet to be proven to exist definitively, with limited evidence to support its validity as a principle of chiropractic care or physiological construct.^24^ There was a marked lack of crossover between articles which examined the historical role of FLS radiography in chiropractic and those which examined the risks associated with the use of FLS radiography (Theme Three), which may be reflective of the contemporary scientific understanding of the risks exposure to ionising radiation which early chiropractic practitioners may not have fully appreciated. Authors were able to draw direct and indirect lineages between chiropractic techniques still practised today and the teachings of BJ Palmer, the patriarch of chiropractic radiology.^2^, ^24^, ^25^ The findings of these articles substantively confirm the use of spinography in the past and by some still in contemporary chiropractic to detect CVS.^2^, ^24^, ^25^

#### Theme two – Clinical indications for FLS radiography

Of the 25 eligible articles, nine described guidelines for the appropriate use of FLS radiography in chiropractic or examined awareness and adherence to those guidelines in practice.^3^, ^4^, ^5^, ^11^, ^12^, ^16^, ^17^, ^18^, ^23^ The Australian Medicare Benefit Schedule Review Taskforce’s committee for diagnostic imaging in low back pain concluded that the clinical indications for FLS radiography are limited primarily to the continuing management of patients with scoliosis, which could typically be managed by a spinal specialist physician as opposed to a chiropractor.^9^ Three authors concluded that chiropractors had low self‐reported knowledge of guidelines for the referral of patients for imaging for low back pain, including the use of FLS radiography.^11^, ^17^, ^18^ One study concluded that only 50% of practising Australian chiropractors had knowledge or awareness of evidence‐based guidelines for imaging in low back pain.^11^ That same study found that up to 20% of chiropractors believe that atraumatic low back pain is an appropriate indication for the referral of patients for FLS radiography,^11^ despite strong evidence suggesting that plain‐film spinal radiography of any region is not indicated in the initial clinical management of this common presentation.^5^, ^11^, ^23^ Authors also described the use of ‘routine’ or repeated radiography of the spine to monitor progressive structural changes.^5^, ^12^ It was largely concluded that repeat radiography of the whole spine should be discouraged as a practice, as it defies current evidence for the appropriate utilisation of spinal radiography.^5^, ^12^

The use of FLS radiography as a screening tool for ‘red flags’ was examined in several papers.^3^, ^5^, ^11^, ^12^ Red flags are underlying spinal pathology which would prevent a chiropractor from being able to safely manipulate a patient’s spine and whose management requirements would exceed their scope of practice, for example metastases, Paget’s disease and infection.^5^, ^12^ The use of FLS radiography as a screening tool for the identification of pathology commonly cited as a red flag by chiropractors without clinical suspicion of disease is discouraged.^5^, ^12^ It was determined that the technical limitations of plain‐film radiography also render these examinations insensitive for the detection of many red flag pathologies, with spinal magnetic resonance imaging and computed tomography deemed more sensitive than FLS radiography.^5^ However, where clinical suspicion of disease which may exceed the scope of chiropractors exists, it could be argued that timely referral to a physician (who may have greater access to MRI and CT) may confer more benefit to patients rather than chiropractic referrals for FLS radiographic investigations.^5^ The sparse evidence surrounding the use of FLS radiography for the screening of red flags both with and without clinical suspicion of underlying pathology brings into question the alignment of these practices with current evidence. Further research is required to determine the scope of this issue as it exists today.

#### Theme three – Risks associated with FLS radiography

This review identified nine eligible articles which addressed this theme.^3^, ^4^, ^5^, ^9^, ^11^, ^12^, ^19^, ^29^, ^30^ There was significant variation between the conclusions drawn by authors about whether exposure to ionising radiation during FLS radiography carried a risk of inducing cancer later in life, with authors advocating for both the liberal and conservative utilisation of FLS radiography by chiropractors.^3^, ^4^, ^5^, ^11^ Two technical studies were identified which quantified effective radiation dose for FLS radiography in AP and lateral projections as well as the likelihood of inducing cancer secondary to undergoing annual FLS radiographic examinations.^29^, ^30^ Technical data also described the limitations of FLS radiography as a radiographic projection, with impacts on the reliability of measurements obtained as part of chiropractic biomechanical analysis techniques.^20^ Aside from the established risks of inducing cancer from FLS radiography, authors also described economic and diagnostic risks associated with the overutilisation of spinal radiography in chiropractic, including waste, false reassurance and overdiagnosis of pathology with limited diagnostic benefit conferred to patients.^5^, ^11^, ^12^ A breakdown of the conclusions authors drew with regards to the risks associated with FLS radiography is included in Table 2.

**Table 2:** Author’s conclusions regarding the risks associated with FLS radiography (Theme Three).

ID Author, Year	Classification of conclusions drawn by authors				Synopsis/supporting evidence
	3a. FLS X‐rays carry a risk of inducing cancer	3b. FLS X‐rays carry a negligible risk or do not carry a risk of inducing cancer	3c. FLS X‐rays carry risks other than cancer, that is economic burden, overdiagnosis	3d. FLS X‐rays are technically limited as a diagnostic investigation	
Coleman, 2011^20^				•	Coleman compared simulated degrees of beam divergence as a determinant of image quality between an anteroposterior (AP) FLS X‐ray at 84 inches focal‐film distance (FFD) and selected sectional spine views at 40 inches FFD.^20^ Coleman described the shortcomings of the AP FLS projection with respect to distortion of anatomical features used for measurement by chiropractors including projected axial rotation and ilium length.^20^ It was determined that the 84‐in full spine view decreased lateral vertebral translation induced y‐axis rotation distortion compared to the 40‐in sectional view, however, the higher focal spot compared to the 40‐in sectional view of the pelvis produced lowering and lengthening of the appearance of the ilium.^20^ [Correction added on 26th January 2022 after first online publication: This point replaces ’It was determined that the distortion was caused by the increased FFD required to obtain an FLS X‐ray.’]
Corso, 2020^12^	•				In a rapid review of 23 articles investigating the clinical utility of routine and repeat radiography of the spine in chiropractic, Corso *‘found no evidence that radiographs used to assess the function or structure of the spine improves patients’ outcomes’* ^12^ Corso argued that radiography of the spine is still indicated for the investigation of ‘red flags’; pathology which would prevent a chiropractor from being able to safely manipulate a patient’s spine, that is metasteses^12^
Harrison, 2018^4^		•			In response to the American Chiropractic Association’s (ACA) endorsement of the ‘Choosing Wisely’ initiative and advocacy for the conservative utilisation of spinal imaging in chiropractic, Harrison argues that restricting spinal imaging, including FLS radiography, puts patient safety at risk and that the position of the ACA is *‘not consistent with the views of large factions of the profession that routinely X‐ray their patients’*.^4^ Although Harrison does not specifically address the risk of inducing cancer from FLS radiography, the author frequently cites articles which argue that there is a non‐existent or negligible risk of inducing cancer from spinal radiography as evidence against the ACA’s position^4^ Harrison is also listed as a contributor in (Oakley, 2020)^3^
Jenkins, 2016^11^	•				In a cross‐sectional survey examining knowledge of and adherence to guidelines for the imaging of atraumatic low‐ back pain by chiropractors (LBP), Jenkins concluded that reported knowledge and adherence to published guidelines amongst Australian chiropractors was low and needed to be improved.^11^ Described a likely association between the use of FLS radiography as prescribed by certain chiropractic techniques, that is Gonstead and Chiropractic BioPhysics in the presence of LBP and poor adherence to guidelines which discourage the use of radiography for the investigation of LBP^11^
Jenkins, 2018^5^	•		•	•	In a narrative review of literature, Jenkins outlined current evidence for the use of spinal radiography in chiropractic, including FLS radiography Jenkins opines that although the risk of inducing cancer from exposure to ionising radiation should not be a barrier to ordering imaging where it is clinically justifiable, *‘it should be assumed that some level of risk is associated with X‐rays’*.^5^ Jenkins also discussed risks aside from inducing cancer, including waste, false reassurance and over/underdiagnosis of pathology and a lack of evidence to support the continued use of spinal radiography as a screening tool for ‘red flags’, citing its lack of specificity for common spinal pathology which would be considered a red flag.^5^
Law, 2016^29^	•				Cumulative organ absorbed doses of repeat AP and lateral FLS X‐rays for progressive scoliosis imaging, performed on an annual basis were simulated and a lifetime attributable cancer risk was calculated as 0.08‐0.17%.^29^ This finding demonstrates a correlation between FLS radiography and the onset of cancer as a stochastic effect of exposure to ionising radiation.^29^
MBS Review Taskforce, 2016^9^	•		•		The Medicare Benefit Schedule (MBS) review taskforce’s diagnostic imaging clinical committee for imaging for LBP concluded that at the time the committee submitted its report (c.2016), there was marked overutilisation of FLS radiography by Australian chiropractors.^9^ The committee noted that three‐ to four‐region spinal X‐rays have limited clinical utility but did concede that they were useful in the assessment of scoliosis. It went on to note that the management of scoliosis would preferentially be undertaken by spinal specialists as opposed to primary care providers, that is chiropractors.^9^
Mogaadi, 2012^30^	•				Mogaadi performed a retrospective analysis of radiation doses encumbered upon patients undergoing FLS radiography as part of the assessment of scoliosis and was able to quantify an effective dose range of between 118 to 1596 μSV for an AP FLS projection and 97 to 1370 μSV for a lateral FLS projection.^30^ Mogaadi stipulated that effective dose is a primary indicator of radiation risk of malignancy.^30^
Oakley, 2020^3^		•			Secondary to Harrison’s refutation of the position of the ACA to endorse the ‘Choosing Wisely’ imaging reduction movement, Oakley asserts that infrequent X‐ray use is not associated with increased risk of cancer and that ‘any guidelines…alluding to dangerous patient radiation exposures as a rationale to avoid imaging is not an evidence‐based argument’ Oakley also disputes key arguments put forth by the ACA and other authors as causation to reduce spinal radiography usage in chiropractic, including economic waste and risks of over and underdiagnosis.^3^

#### Theme four – Chiropractic techniques which prescribe the use of FLS radiography

A total of 13 articles described the use of FLS radiography as prescribed by contemporary chiropractic techniques or treatment systems.^2^, ^3^, ^4^, ^5^, ^6^, ^11^, ^12^, ^15^, ^20^, ^24^, ^25^, ^26^, ^27^, ^28^ Six published techniques were identified which require the use of FLS radiography including the *Gonstead, Chiropractic BioPhysics, Applied Spinal Biomechanical Engineering, Universal Chiropractic College, Pierce/Stillwagon, Spinal Stressology* and *Logan* techniques.^4^, ^5^, ^12^, ^24^ Authors described a strong correlation between the use of these techniques and increased reliance on FLS radiography, suggesting incongruity between adherence to guidelines for spine imaging and the pressure placed on chiropractic practitioners to adhere to the requirements of a given technique system.^11^, ^12^ As such, the use of spinal radiography as part of a specific technique may be a barrier to the appropriate use of FLS radiography in practice.^5^, ^11^ There is a lack of high‐quality evidence to suggest that radiographic measurements taken from FLS X‐rays as part of treatment following a chiropractic technique system such as the Chiropractic BioPhysics system are clinically relevant or have any bearing in the outcomes afforded to patients.^12^ This review did not examine the effectiveness or credibility of techniques which use FLS radiography and or the role of FLS radiography as a part of individual chiropractic techniques. Indications for the use of techniques requiring FLS radiography in practice were also not examined; however, it is apparent that the use of chiropractic techniques which incorporate FLS radiography can precipitate the use of FLS radiography where it is otherwise not indicated.^5^, ^12^, ^24^

#### Theme five – Current trends in the utilisation of FLS radiography by chiropractors

This review identified 14 eligible articles which addressed this theme.^2^, ^3^, ^4^, ^5^, ^8^, ^9^, ^11^, ^12^, ^17^, ^18^, ^21^, ^22^, ^23^ The strength of evidence authors used to draw conclusions about the reliance of chiropractors on FLS radiography was lacking. Measures used by authors to determine referral patterns through survey responses were largely self‐reported or framed in a hypothetical context.^11^, ^21^, ^26^, ^27^ Retrospective studies which examined the use of FLS radiography in a given population of chiropractic practitioners suggested differences in education, the availability of medical imaging equipment and knowledge of diagnostic imaging guidelines as determinants of utilisation.^5^, ^11^, ^12^ This review also examined changes made to the Medicare Benefit Schedule (MBS) in 2017, which limited the ability of chiropractors to refer patients for MBS‐reimbursed FLS radiography.^8^, ^9^ These changes were made after a taskforce determined that FLS radiography was being overutilised by chiropractic referrers and was implemented ostensibly to dissuade reliance on this examination.^8^, ^9^ However, the changes did not remove the ability of chiropractors to order FLS X‐rays entirely and today chiropractors are still able to order FLS radiographic examinations which do not attract a Medicare benefit.^8^ The introduction of these changes also removed the only reliable measure of the number of FLS X‐rays being performed in Australia today; a measure which was previously used as a cause for reform.^9^ Therefore, it is difficult to determine whether the changes enacted in 2017 were effective in their goal of reducing the number of FLS X‐rays ordered by chiropractors.

An additional six articles were identified which also examined current trends in the availability and utilisation of medical imaging equipment used for full‐length spinal radiography by chiropractors.^3^, ^5^, ^6^, ^8^, ^9^, ^11^ As part of their clinical training, chiropractors are trained in general radiographic techniques and can obtain radiation usage licenses for the operation of radiographic equipment in their practices.^5^, ^11^ The ownership of radiographic equipment by chiropractors was determined to be a possible cause of increased utilisation of FLS radiography in practice.^5^ It remains unclear what proportion of chiropractors opt to perform their own radiography or otherwise preferentially refer their patients to medical imaging practices for the completion of a spinal X‐ray series. It is also unclear how the decision to refer patients to medical imaging practices for these series impacts the completion of the examination itself and how clinical justification is assessed in this setting, given the specialist nature of chiropractic techniques which could be considered esoteric.^5^, ^6^, ^11^ Further research is required to understand the dynamics at play in the completion of chiropractor‐requested FLS radiography by radiographers in medical imaging practices.

## Discussion

The results of this review point to the existence of a spectrum of beliefs and knowledge amongst chiropractic practitioners surrounding the appropriate use of FLS radiography which may not always align with the principles of evidence‐based practice.^2^, ^8^, ^9^, ^11^, ^17^, ^18^, ^21^ It should be noted from the outset that in many ways, the issue of inappropriate or unnecessary referrals for medical imaging and other diagnostic investigations is not an issue which is exclusive to chiropractors.^9^, ^10^, ^31^ In the context of the wider discussions surrounding the appropriate use of medical imaging, particularly spinal imaging, overutilisation and departures from established imaging guidelines have been reported extensively amongst medical practitioners.^9^ As such, the issue at hand is not whether overutilisation of FLS radiography can be attributed to chiropractors as alternative health practitioners or chiropractic as a discipline.^3^, ^4^ Rather, the issue, which is underscored by the findings of this review, is how health professionals can work collaboratively to ensure the judicious, evidence‐based use of FLS radiography which better aligns with the delivery of quality health outcomes for patients.

The risks associated with overutilisation of diagnostic imaging are well documented.^5^, ^8^, ^11^, ^12^, ^17^, ^21^, ^29^, ^30^ Aside from the inherent risks of unnecessary exposure to ionising radiation, increased reliance on diagnostic imaging by any practitioner in the absence of sufficient clinical justification increases economic burdens encumbered upon the health care system. As such, FLS radiography should be used judiciously to ensure risks associated with its use are minimised, thus ensuring that it remains available to chiropractors and other practitioners where its use is clinically justified.^7^, ^9^ Imaging which is not clinically indicated also carries a risk of overdiagnosis that being the radiological diagnosis of disease which does not ultimately impact on a patient’s course of treatment.^5^ In a narrative review, Jenkins concluded that in chiropractic applications of spinal radiography, this could include the diagnosis of incidental degenerative changes such as osteophyte formation, reduced disk height and spondylolisthesis.^5^ These findings are common, but show poor correlation with clinical symptoms and render no benefit for patients undergoing treatment.^5^ Furthermore, the reported use of FLS radiography by chiropractors for the detection of red flags in the absence of any significant clinical indications for imaging could also be considered a practice which also carries a high risk of overdiagnosis.^5^, ^11^, ^12^, ^18^ Ironically, overutilisation of FLS radiography may also lead to an increased risk of missed or underdiagnosis, a failure to detect early pathological changes due to the insensitivity of a diagnostic test (Type II error).^5^ Plain film X‐rays have a significant insensitivity to early structural changes in bone, suggestive of an acute pathology and as such the continued use of FLS radiography to identify changes in this nature as part of screening for red flags may not be consistent with current evidence.^5^

For radiographers, the completion of imaging which is clinically unjustified stands as a challenge to their professional responsibilities as health practitioners, and the guiding principles of radiation safety.^10^, ^31^ In practice, radiographers must ensure that the imaging examination they have been asked to perform by a referring practitioner is appropriate in the context of the clinical information and the known risks of exposure to ionising radiation.^10^, ^31^ This responsibility is aligned with the ALARA (As Low as Reasonably Achievable) principle of radiation safety, which stipulates that the use of ionising radiation should be conservative, clinically appropriate and justified when weighed against the known risks of exposure to ionising radiation, namely the risk of inducing cancer later in life.^5^, ^10^, ^31^ The wider responsibility to assess whether the risks of a patient undergoing radiographic imaging outweigh the diagnostic benefit of the examination lies primarily with the referring practitioner who instigates a request for imaging; however, radiographers have an important role to play in the assessment of clinical justification in medical imaging.^10^, ^31^ Barriers to the assessment of clinical justification by radiographers have been described including differences in education and medical dominance.^10^ These barriers are compounded in the case of chiropractor‐requested FLS radiography, by a lack of shared knowledge which could be bridged with further research and improved dialogue with chiropractic practitioners.

**Figure 2:**
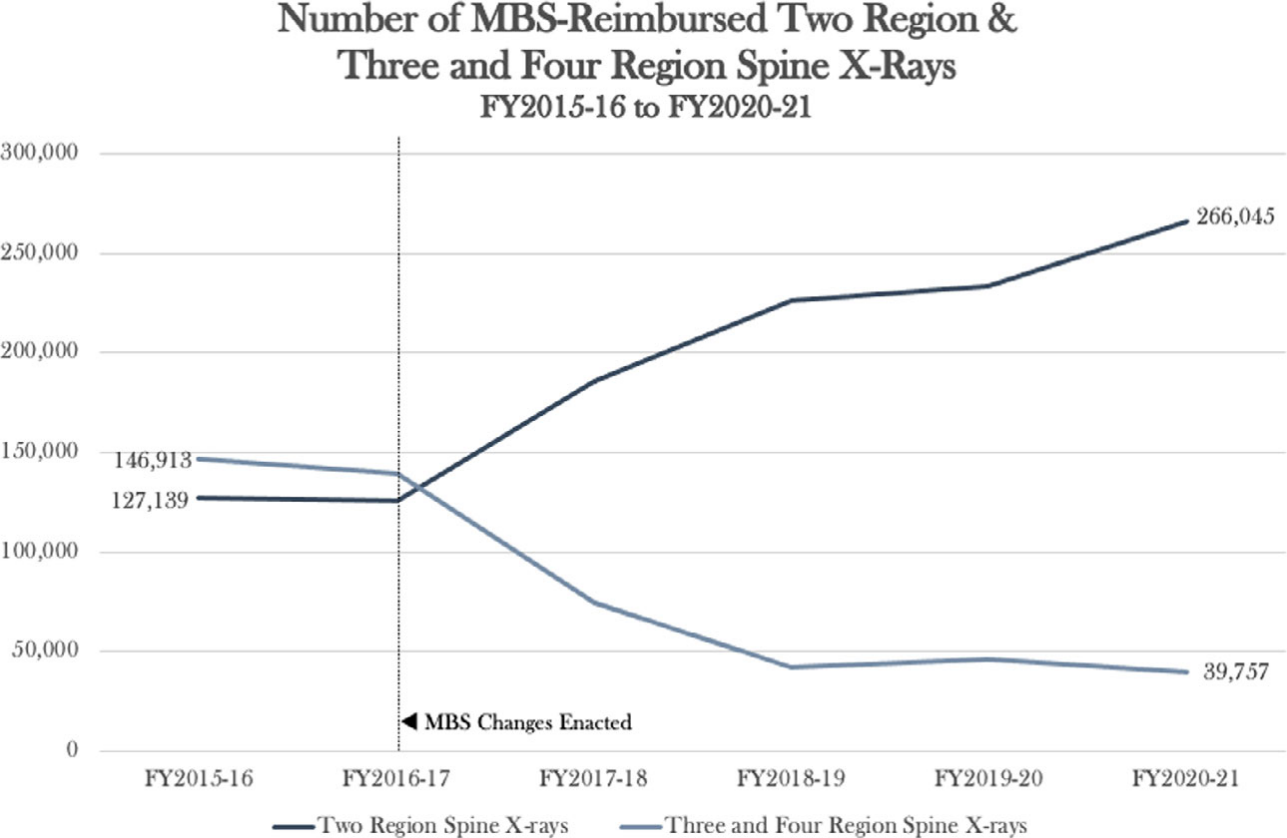
Number of MBS‐reimbursed two‐region and three‐ and four‐region Spine X‐rays (FY2015‐16 to FY2020‐21).^32^, ^33^ Permission was obtained to reproduce this figure.

In the context of FLS radiography utilisation by chiropractors, overutilisation has previously been reported and may still be prevalent today.^8^, ^9^ The introduction of the 2017 changes to MBS reimbursement for three‐ and four‐region spinal radiography have precipitated a substantial decline in the number of MBS‐compensated three and four‐region spinal X‐rays completed in the four years since the changes were enacted.^32^ By comparison in the same period, the number of MBS‐reimbursed two‐region spinal X‐rays completed rose dramatically, as shown in Figure 2.^33^

The relevance of this finding in the context of chiropractor utilisation of FLS radiography is unclear. These data reflect only MBS‐reimbursed examinations, which as a direct result of the 2017 MBS changes no longer includes referrals made by chiropractors.^8^, ^32^ Chiropractors are still able to request FLS X‐rays; however, they attract no MBS benefit, with the cost of the examination shifted to patients themselves or otherwise absorbed by the imaging service provider.^9^ In their final report to the MBS review taskforce, the committee for medical imaging in low back pain argued that mechanisms which would effectively bypass the regulatory changes enacted by the MBS review taskforce could exist and should be closed to ensure the goals of the changes were met.^9^ This could possibly include incorrect choice of billing codes, that is billing for a two‐region spinal X‐ray instead of a three‐ or four‐region examination.^9^ The existence of such loopholes may explain the increased volume of MBS‐reimbursed two‐region spinal X‐rays completed since FY2016‐17^33^; however, these changes could also be consistent with population growth and a general trend of increased utilisation of diagnostic services. It would exceed the scope of this review to explore these mechanisms; however, investigation is required to determine whether the taskforce’s changes were effective in their goal of reducing unnecessary referrals for FLS radiography by chiropractors.^8^

Evidently, the issues outlined in this review are important and are worthy of consideration by both chiropractors and radiographers. There is both the scope and room for the two professions to work collaboratively in ensuring the clinically appropriate use of FLS radiography as a way of reducing unnecessary investigations and improving patient outcomes. The authors recognise the limitations of this scoping review process, namely a lack of critical review of the quality of evidence examined in the review and the broad scope in which the review was framed.^34^ It is also acknowledged that the decision to limit eligibility to articles published between 2010 and 2021 leads to a substantial reduction in the volume of evidence which was able to be drawn upon. The strengths of this scoping review are in its broad scope, the comprehensive search strategy employed to identify current evidence and its satisfaction of its aims, which were principally to outline the various facets of FLS radiography use in chiropractic as it exists today. Further investigation is required to more definitively address this issue moving forward.

## Conclusion

This review has identified a scarcity of literature addressing the completion of chiropractor‐referred FLS X‐rays. Our review has outlined several pressing issues that warrant further investigation including a lack of quantitative measures to assess the utilisation of FLS X‐rays by chiropractors, a lack of consensus of what constitutes appropriate clinical justification for imaging and the existence of a spectrum of beliefs amongst chiropractic authors about the clinical utility and limitations of FLS radiography. This provides radiographers with a definitive opportunity to demonstrate clinical leadership in this space and seek to begin a constructive dialogue with chiropractic referrers about the risks associated with unnecessary or unjustified spinal radiography. In doing this, diagnostic radiographers as evidence‐based health practitioners can actively contribute to the conversation surrounding the issues identified by this study and can be better positioned to advocate for the interests of the discipline and the safety of their patients.

## Funding Sources

No funding was received for this project. Access to commercial databases was provided by the University of Sydney.

## Conflicts of Interest

No conflicts of interest are declared by the authors.
